# ARD1 contributes to IKKβ-mediated breast cancer tumorigenesis

**DOI:** 10.1038/s41419-018-0921-2

**Published:** 2018-08-28

**Authors:** Yu Zhang, Hang Zhou, Yongjun Tao, Xingyu Liu, Zhu Yuan, Chunlai Nie

**Affiliations:** 1 Department of Oncology, Guizhou Provincial People’s Hospital, 550002 Guizhou, China; 2 Department of Chemotherapy, Sichuan Cancer Hospital & Institute, Sichuan Cancer Center, School of Medicine, University of Electronic Science and Technology of China, 610041 Chengdu, China; 3 People’s Hospital of Danzhai County, 557500 Guizhou, China; 4 State Key Laboratory of Biotherapy and Cancer Center, West China Hospital, Sichuan University and Collaborative Innovation Center for Biotherapy, 610041 Chengdu, China

## Abstract

The expression of IκB kinase β (IKKβ) promotes the growth of breast cancer cells. Meanwhile, IKKβ mediates the phosphorylation and subsequent degradation of arrest-defective protein 1 (ARD1). However, the relationship between IKKβ and ARD1 in the occurrence of breast cancer has not been reported. In this study, we found that IKKβ not only acts directly on mammalian target of rapamycin (mTOR) activity but also indirectly acts on mTOR activity through posttranscriptional modification of ARD1, thereby effectively promoting the growth of breast cancer cells. ARD1 prevents mTOR activity and breast cancer cell growth by stabilizing tuberous sclerosis complex 2 (TSC2) to induce autophagy. Moreover, acetylation of heat shock protein 70 (Hsp70) also contributes to ARD1-mediated autophagy. Therefore, upstream IKKβ can further promote the occurrence of breast cancer by mediating the function of ARD1.

## Introduction

IκB kinase β (IKKβ) is an integral part of the IKK complex. The complex consists of IKKα, IKKβ, and a regulatory subunit, IKKγ^1–4^. IKKβ is a major downstream kinase in the tumor necrosis factor α (TNFα) pathway^5^ and can be activated by inflammatory signals such as TNFα or lipopolysaccharide (LPS). Activated IKKβ can promote the nuclear translocation of nuclear factor κB (NF-κB) by phosphorylation and degradation of IκBα^1,4,6^. In the nucleus, NF-κB activates its target genes to initiate a series of functions. Constitutive activation of IKK and NF-κB family members contributes to the development of breast cancer^3^. Previous studies showed that IKKβ promoted the development of breast carcinoma by phosphorylating two tumor suppressor factors, forkhead box O3a (FOXO3a) and tuberous sclerosis complex 1 (TSC1). IKKβ starts the ubiquitin degradation pathway of FOXO3a and TSC1, inhibiting the function of the two factors and promoting the occurrence of breast cancer^2,5^.

Arrest-defective protein 1 (ARD1; also known as N-α-acetyltransferase 10 [Naa10p]) was originally found in yeast and is a catalytic subunit of the NatA acetyltransferase, which is responsible for N-terminal α-acetylation^7,8^. ARD1 has both N-terminal α-protein and ε-protein acetyltransferase activities, and promotes the growth of lung cancer cells through the ε-acetylation of β-catenin^8,9^. A previous study revealed that ARD1 overexpression correlated with poor survival of human lung cancer patients^10^. ARD1 was found to be overexpressed in breast cancer^11^, colorectal cancer^12^, and hepatocellular cancer^13^. Likewise, ARD1 also mediates the growth of colon cancer cells, and high expression of ARD1 in colon cancer is associated with poor prognosis^12,14^. Depletion of ARD1 sensitizes colon cancer cells to induce apoptosis through RelA/p65-regulated MCL1 expression^15^. These findings tend to support the model that ARD1 is an oncoprotein that promotes tumor growth. However, ARD1 was also shown to promote DNA damage-mediated apoptosis^8,16^. Furthermore, ARD1 was found to inhibit breast and lung cancer cell metastasis^17–19^. Meanwhile, increased ARD1 expression was reported to associate with better clinical effects in patients with breast and lung cancer. ARD1 overexpression inhibited breast cancer cell growth and tumorigenesis^17–19^. These results suggest that ARD1 may function as a tumor suppressor. These conflicting experimental data might result not only from different experimental methods and materials in different laboratories but also might indicate that ARD1 can play different roles in different tumor cell types and even subtypes. After all, ARD1 is highly expressed in primary tumors but has low expression in tumors with lymph node metastases^17^.

In this study, we further explored the pathway of IKKβ-mediated tumorigenesis. We first found that ARD1 overexpression decreased IKKβ-mediated breast cancer tumorigenesis. As described in a previous report^6^, our data also demonstrated that IKKβ phosphorylated and then degraded ARD1 in breast cancer cells. Mutation of the IKKβ phosphorylation site in ARD1 affected the growth of IKKβ-mediated tumor cells. Further experiments revealed that ARD1 restrained the occurrence of IKKβ-mediated breast cancer by inducing autophagy. Moreover, we found that ARD1 mediated autophagy by two signaling pathways. In the first pathway, ARD1 inhibits mammalian target of rapamycin (mTOR) activity to increase autophagy by stabilizing tuberous sclerosis complex 2 (TSC2) as described previously^19^. In the second pathway, ARD1 mediates heat shock protein 70 (Hsp70) acetylation to promote autophagy. In this way, in addition to inhibiting the function of TSC1^5^, IKKβ also promotes the growth of breast cancer by acting on ARD1.

## Results

### IKKβ-mediated ARD1 degradation is required for IKKβ-induced growth of breast cancer cells

**Fig. 1:**
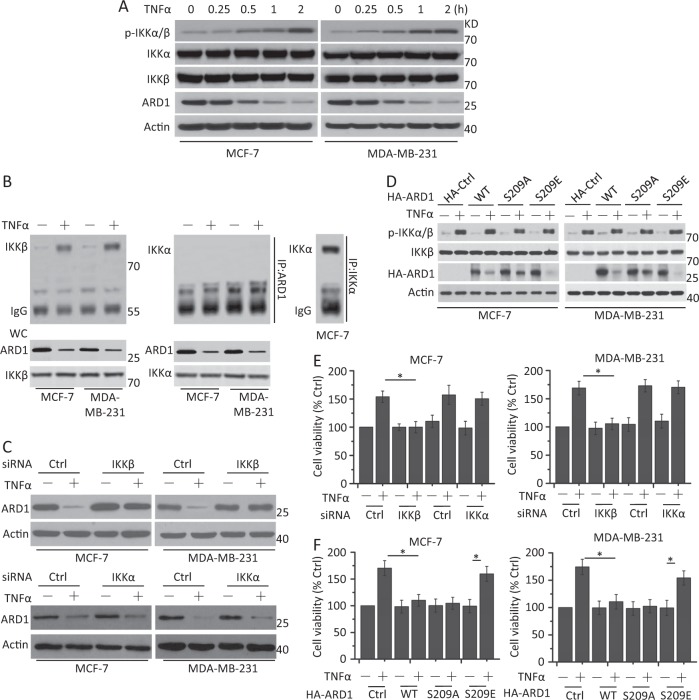
ARD1 mediates TNFα-induced breast cancer cell growth through IKKβ-induced phosphorylation. **a** MCF-7 and MDA-MB-231 cells were serum-starved overnight and treated with TNFα (10 ng/ml) at indicated time, and then cells were collected for western blot analysis of the expression of the protein shown in the figure. β-Actin was used as a protein loading control. p-IKKα/β means the phosphorylation status of IKKα/β. **b** ARD1 physically interacted with IKKβ in breast cancer cells after TNFα treatment. Cells were serum-starved overnight, and then treated with TNFα (10 ng/ml) for 30 min and cell lysates were immunoprecipitated with anti-ARD1 and immunoblotting by anti-IKKβ antibodies. IKKα coimmunoprecipitation and immunoblotting assays were as a control. WC means whole lysates. **c** Cells were transfected with Ctrl siRNA, IKKβ, or IKKα siRNA for 48 h, and then serum-starved overnight and treated with TNFα for 1 h. Treated cells were collected for western blot analysis of the expression of the protein shown in the figure. β-Actin was used as a protein loading control. **d** Cells were transfected with HA-Ctrl, HA-ARD1 wild type (WT), or HA-ARD1 mutants for 48 h, serum-starved overnight, and then treated with TNFα (10 ng/ml) for 1 h. Treated cells were collected for western blot analysis of the expression of the protein shown in the figure. **e** Cells were transfected with Ctrl siRNA, IKKβ, or IKKα siRNA for 48 h, serum-starved overnight, and then treated with TNFα for 1 h. Cell viability and proliferation was determined as CellTiter-Glo Luminescent Cell Viability Assay. Graphs showing results of quantitative analyses (*n* = 3, mean ± S.D. *P < 0.05). **f** Cells were transfected with HA-Ctrl, HA-ARD1 WT, or HA-ARD1 mutants for 48 h, serum-starved overnight, and treated with TNFα for 1 h. Cell viability and proliferation was determined as **e**. Graphs showing results of quantitative analyses (*n* = 3, mean ± S.D. *P < 0.05)

We first examined protein expression after TNFα treatment. We found that the phosphorylation levels of IKKα and IKKβ were increased in a time-dependent manner. There was little change in the total expression of IKKα and IKKβ. Meanwhile, ARD1 expression was decreased after TNFα treatment (Fig. 1a). We then used the protease inhibitor MG132 and TNFα in combination to treat the cells. Our data showed that the decreased ARD1 expression was suppressed (Supplementary Fig. 1A), indicating that ARD1 was degraded after TNFα treatment.

Previous work has revealed that IKKβ interacts with ARD1 and mediates its phosphorylation and degradation^6^. We therefore examined whether IKKβ interacts with ARD1 after TNFα treatment. As depicted in Fig. 1b, IKKβ interacts with ARD1 in breast cancer cells after TNFα treatment, while IKKα does not. We then cotransfected Flag-IKKβ, Flag-IKKα, or kinase-dead mutants of IKKβ and IKKα, which carry a K44A mutation (Flag-nIKKβ and Flag-nIKKα), with HA-ARD1 vectors into HEK293T cells. An immunoprecipitation assay revealed that Flag-IKKβ, but not Flag-IKKα, interacted with HA-ARD1 (Supplementary Fig. 1B). Meanwhile, the deficiency of IKKβ kinase activity did not affect the interaction between IKKβ and ARD1 (Supplementary Fig. 1B). An RNAi assay demonstrated that IKKβ depletion, but not IKKα depletion, rescued ARD1 expression (Fig. 1c and Supplementary Fig. 1C).

Previous research has demonstrated that IKKβ phosphorylates ARD1 at its Ser209 site and that mutation of Ser209 affects the phosphorylation levels and degradation of ARD1^6^. We also constructed HA-ARD1 wild type (WT), HA-ARD1 S209A (substitution of Ala for Ser209, nonphosphorylatable mutant), and HA-ARD1 S209E (substitution of Glu for Ser209, phosphorylation-mimic mutant) as described before^6^. We then transfected the different ARD1 vectors into cells and found that the ARD1 mutants did not affect the activity of IKKβ or IKKα, indicating that IKKβ is the upstream regulator of ARD1. The degradation levels of ARD1 between the HA-ARD1 WT and the S209A mutants were not significantly different in the presence of TNFα treatment. However, the HA-ARD1 S209E mutant promoted a decrease in ARD1 abundance (Fig. 1d). These results demonstrated that TNFα-induced IKKβ activation contributes to the phosphorylation and degradation of ARD1 in breast cancer cells.

We then measured the growth of breast cancer cells. IKKβ depletion, but not IKKα depletion, indeed decreased cell growth in the presence of TNFα (Fig. 1e) as described before^5^. Exogenous HA-ARD1 and HA-ARD1 S209A expression decreased cell growth after TNFα treatment; however, HA-ARD1 S209E had little effect on the growth of breast cancer cells after TNFα treatment (Fig. 1f). These results indicated that residual, exogenous ARD1 WT or S209A restrained cell growth, although HA-ARD1 and HA-ARD1 S209A were degraded (Fig. 1d), further confirming the inhibitory effect of ARD1 on cell growth. Meanwhile, our data also revealed that HA-ARD1 and HA-ARD1 S209A, but not S209E, restrained IKKβ-mediated cell growth (Supplementary Fig. 1D). These results demonstrated that IKKβ-mediated ARD1 phosphorylation and degradation is required for IKKβ-induced cell growth.

### IKKβ-mediated ARD1 degradation promotes mTOR activity by TSC2

**Fig. 2:**
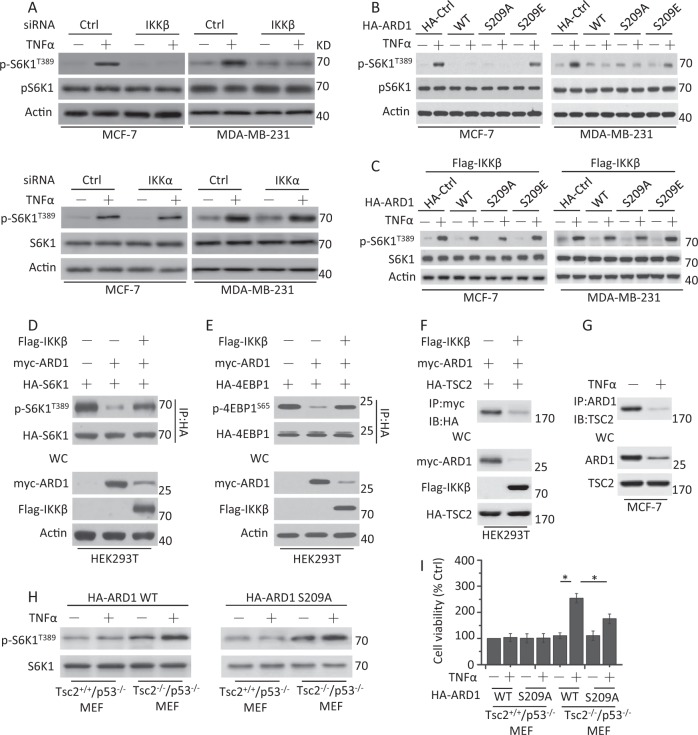
ARD1 inhibits the mTOR activity through IKKβ regulation. **a** Cells were transfected with Ctrl siRNA, IKKβ, or IKKα siRNA for 48 h, serum-starved overnight, and then treated with TNFα for 1 h. Treated cells were collected for western blot analysis of the expression of the protein shown in the figure. β-Actin was used as a protein loading control. p-S6K1^T389^ means the phosphorylation status of S6K1. **b** Cells were transfected with HA-Ctrl, HA-ARD1 WT, or HA-ARD1 mutants for 48 h, serum-starved overnight, and treated with TNFα for 1 h. Treated cells were collected for western blot analysis of the expression of the protein shown in the figure. **c** Cells were cotransfected with FLAG-IKKβ, HA-Ctrl, HA-ARD1 WT, or HA-ARD1 mutants for 48 h, serum-starved overnight, and treated with TNFα for 1 h. Treated cells were collected for the detection of protein expression. **d** Lysates of HEK293T cells cotransfected with FLAG-IKKβ, Myc-ARD1, and HA-S6K1 were analyzed with antibodies directed against the HA tag by coimmunoprecipitation and immunoblotting by p-S6K1^T389^ and HA. **e** HEK293T cells were cotransfected with FLAG-IKKβ, Myc-ARD1, and HA-4EBP1, and then the cells were lysed for coimmunoprecipitation assay as described in **d**. **f** HEK293T cells were cotransfected with FLAG-IKKβ, Myc-ARD1, and HA-TSC2 and then treated as described in **d**. **g** MCF-7 cells were serum-starved overnight and treated with TNFα for 1 h. Cell lysates were immunoprecipitated with specific antibodies to ARD1 and TSC2 to identify the association of endogenous proteins. **h** Indicated cells were transfected with HA-ARD1 WT or HA-ARD1 S209A, serum-starved overnight, and then treated with TNFα for 1 h. Cell lysates were collected for the detection of the change of p-S6K1^T389^. **i** Treated cells as described in **h** were collected for the detection of cell viability and proliferation. Graphs showing results of quantitative analyses (*n* = 3, mean ± S.D. *P < 0.05)

Previous work has demonstrated that IKKβ promotes mTOR activity^5^. Our data also revealed that the phosphorylation levels of S6K1 at T389 (p-S6K1^T389^), which is a well-known mTOR phosphorylation site, increased in breast cancer cells with TNFα treatment. IKKβ depletion, but not IKKα depletion, indeed decreased the phosphorylation levels of S6K1, suggesting that mTOR activation is inhibited (Fig. 2a).

We then found that ARD1 depletion had little effect on the phosphorylation levels of S6K1 in untreated cells. Meanwhile, the phosphorylation levels of S6K1 were almost unchanged after TNFα treatment (Supplementary Fig. 2A). However, HA-ARD1 and its nonphosphorylatable mutant HA-ARD1 S209A restrained the phosphorylation levels of S6K1, while the phosphorylation-mimic mutant HA-ARD1 S209E increased S6K1 phosphorylation in TNFα-treated cancer cells (Fig. 2b). Further experiments revealed that Flag-IKKβ rescued S6K1 phosphorylation in the presence of HA-ARD1 and its mutants (Fig. 2c). An immunoprecipitation assay also demonstrated that the phosphorylation levels of S6K1 or 4EBP1 at S65 (another well-known mTOR phosphorylation site) were partially restored by IKKβ overexpression (Fig. 2d, e). These results revealed that IKKβ regulates ARD1 to mediate mTOR activation.

ARD1 was reported to interact with TSC2 to suppress mTOR activity^19^. We found that ARD1 indeed interacted with TSC2 in cells by an immunoprecipitation assay, while IKKβ decreased the interaction (Fig. 2f). MG132 treatment restored the interaction between ARD1 and TSC2 in the presence of IKKβ (Supplementary Fig. 2B). TNFα treatment also inhibited the binding of ARD1 and TSC2 (Fig. 2g). Meanwhile, the addition of MG132 maintained the interaction between ARD1 and TSC2 after TNFα treatment (Supplementary Fig. 2C). We next transfected HA-ARD1 WT and S209A into Tsc2^+/+^p53^−/−^ and Tsc2^−/−^p53^−/−^ mouse embryonic fibroblasts (MEFs) (Supplementary Fig. 2D) to determine if ARD1 regulates mTOR through TSC2. The abundance of pS6K1 phosphorylation was decreased in ARD1 WT or S209A-bearing Tsc2^+/+^p53^−/−^ MEFs but not in ARD1 WT or S209A-bearing Tsc2^−/−^p53^−/−^ MEFs with or without TNFα treatment (Fig. 2h). Further experiments revealed that deletion of TSC2 promoted cell growth in ARD1 WT or S209A transfected MEFs. The growth of the ARD1 S209A-bearing cells was lower than that of the ARD1 WT-bearing cells, as ARD1 S209A, which cannot be phosphorylated and degraded by IKKβ, reduced cell growth to some extent (Fig. 2i). These results indicated that ARD1 indeed acts on TSC2 to regulate mTOR activity, further mediating cell growth in our experimental system.

### ARD1 decreases mTOR activity to promote autophagy

**Fig. 3:**
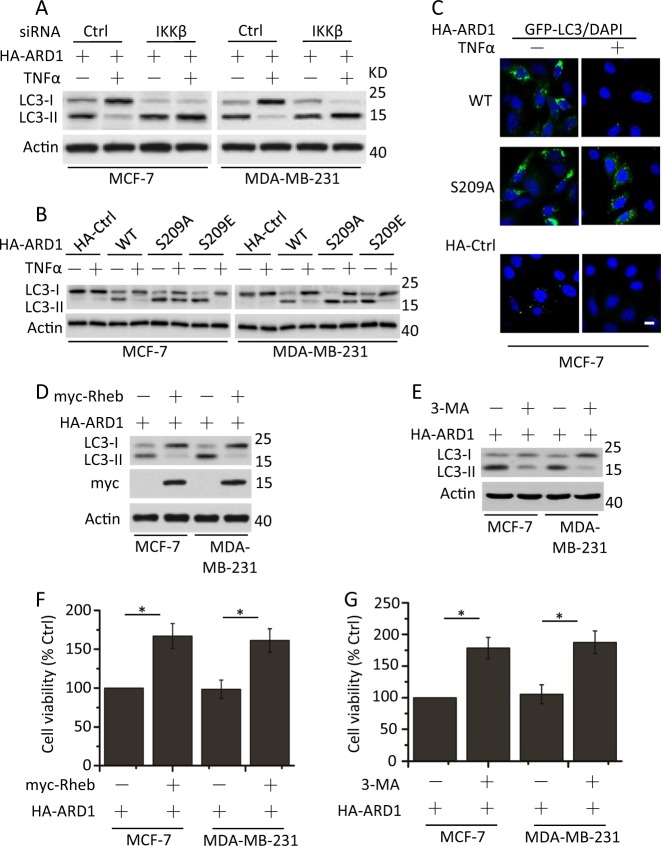
ARD1 restrains mTOR activity to promote autophagy through regulation of IKKβ. **a** MCF-7 and MDA-MB-231 cells were cotransfected with HA-ARD1, Ctrl siRNA, or IKKβ siRNA for 48 h, serum-starved overnight, and then treated with TNFα for 1 h. Cell lysates were collected for detection of LC3-I and LC3-II expression. β-Actin was used as a protein loading control. **b** Cells were transfected with HA-Ctrl, HA-ARD1 WT, or HA-ARD1 mutants, serum-starved overnight, and then treated with TNFα for 1 h. Treated cells were determined as described in **a**. **c** MCF-7 cells were cotransfected with GFP-LC3 and HA-Ctrl, HA-ARD1 WT, or HA-ARD1 S209A, serum-starved overnight, and then treated with TNFα for 1 h. Treated cells were fixed, and then visualized by fluorescent microscopy. Bars, 10 µM. **d** MCF-7 and MDA-MB-231 cells were cotransfected with HA-ARD1 and myc-Rheb. Cell lysates were collected for LC3-I and LC3-II expression detection. **e** Cells were transfected with HA-ARD1 and then treated with the inhibitor of class III PI3 kinases 3-MA (5 mM) for 4 h, lysed and subjected to western blotting with anti-LC3 antibodies to monitor autophagy. **f** and **g** Cells were treated as described in **d** and **e**, respectively. Cell viability and proliferation were determined. Graphs showing the results of quantitative analyses (*n* = 3, mean ± S.D. *P < 0.05)

TNFα-induced activation of IKKβ results in mTOR activation^5^, suggesting that IKKβ contributes to the inhibition of autophagy^20^. Meanwhile, ARD1 expression induces autophagy to decrease cell growth in breast cancer cells^19^. We next examined the relationship between IKKβ, ARD1, and autophagy after TNFα treatment. IKKβ deficiency had little effect on LC3-I and LC3-II expression in TNFα-treated breast cancer cells (Supplementary Fig. 3A); however, exogenous ARD1 expression significantly increased LC3-II expression in TNFα-untreated cells. TNFα treatment decreased ARD1-mediated expression of LC3-II, while IKKβ depletion restored LC3-II abundance regardless of TNFα treatment (Fig. 3a).

We then found that HA-ARD1 S209A-bearing cells resisted the decrease of LC3-II expression compared with HA-ARD1 WT and S209E-bearing cells after TNFα stimulation (Fig. 3b). Meanwhile, in contrast to HA-ARD1 WT, HA-ARD1 S209A maintained the GFP-LC3 puncta after TNFα treatment (Fig. 3c). These results demonstrated that the phosphorylation status of ARD1 regulates LC3 expression and autophagy.

We then tested whether mTOR activation limits LC3-II expression. Overexpression of Rheb, an mTORC1 activator^21^, decreased LC3-II expression in ARD1-bearing cells (Fig. 3d). Meanwhile, Rheb overexpression had little effect on LC3-II expression in cells lacking ARD1 (Supplementary Fig. 3B). 3-Methyladenine (3-MA), which is the most commonly used pharmacological inhibitor of autophagy that blocks the formation of autophagosomes^22^, decreased ARD1-induced autophagosome formation in breast cancer cells as measured by LC3 conversion in ARD1-bearing cells (Fig. 3e). The addition of 3-MA had little effect on the expression of LC3 in cells lacking ARD1 (Supplementary Fig. 3C). Rheb overexpression or 3-MA treatment promoted the growth of ARD1-bearing breast cancer cells (Fig. 3f, g). Meanwhile, Rheb overexpression obviously increased the growth of Ctrl or ARD1 siRNA cells (Supplementary Fig. 3D). 3-MA treatment had little effect on the growth of Ctrl or ARD1 siRNA cells (Supplementary Fig. 3E). However, ARD1 siRNA indeed promoted the growth of breast cancer cells, as described before^19^. These results confirmed that ARD1 indeed inhibits mTOR activity to promote autophagy and decrease the growth of breast cancer cells^19^, which is regulated by IKKβ phosphorylation.

### ARD1 mediates Hsp70 acetylation to promote autophagy

**Fig. 4:**
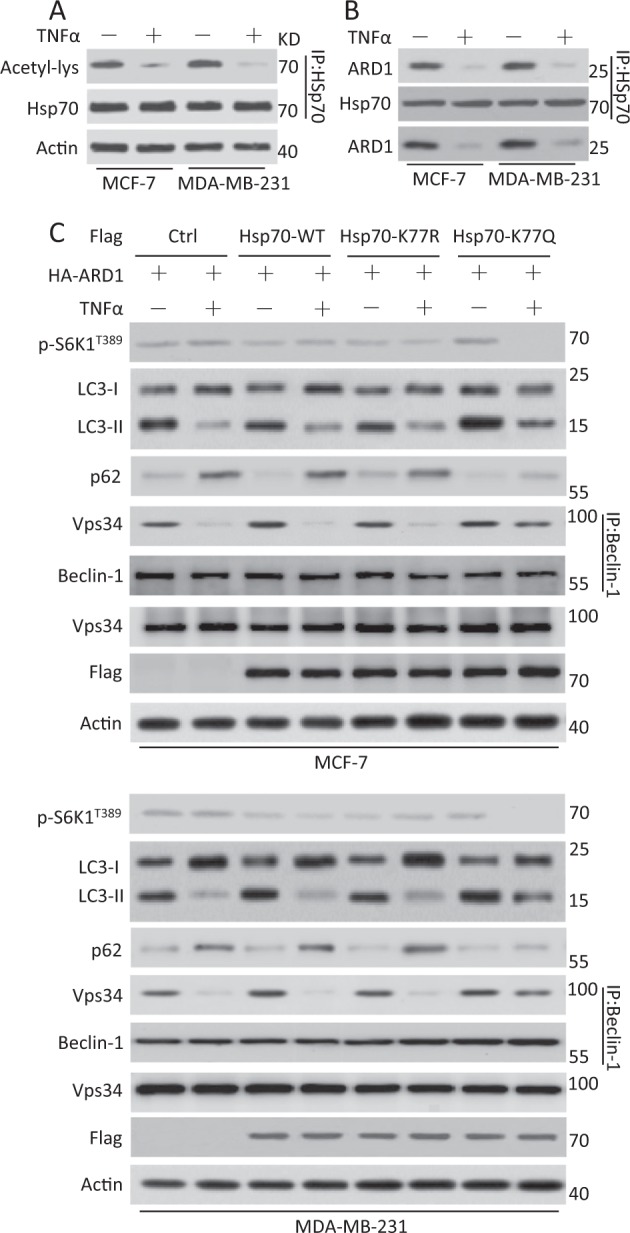
ARD1 mediates Hsp70 acetylation to increase autophagy. **a** Cells were serum-starved overnight and treated with TNFα for 30 min. Protein acetylation was determined by immunoprecipitation with anti-Hsp70 antibody followed by western blotting for an antibody recognizing internal acetyl-lysine residues or anti-Hsp70 antibody. β-Actin was used as a protein loading control. **b** Cells were treated as described in (**a**) and lysates were detected by immunoprecipitation as described in the figure. **c** Cells were transiently cotransfected with HA-ARD1 or Flag Ctrl, Flag vector, Flag-Hsp70 WT, Flag-Hsp70 mutants, serum-starved overnight, and then treated with TNFα for 1 h. Cell lysates were lysed for the detection of the expression of the protein shown in the figure. The association between Vsp34 and Beclin-1 was analyzed by immunoprecipitation with anti-Beclin-1 antibody followed by western blotting for anti-Vsp34 or anti-Hsp70 antibodies

Hsp70 acetylation is required to induce autophagy^23^. Moreover, Hsp70 acetylated by ARD1 protects cells again stress^24^. Hsp70 deacetylation decreases autophagy and cell protection. We next tested whether TNFα could decrease Hsp70 acetylation. We found that Hsp70 acetylation was clearly decreased after TNFα treatment (Fig. 4a). Moreover, Hsp70 interacted with ARD1 in untreated cells and TNFα treatment inhibited this interaction (Fig. 4b). ARD1 depletion further decreased the acetylation of Hsp70 and the interaction between Hsp70 and ARD1 in untreated cells (Supplementary Fig. 4A). However, the addition of MG132 restored the binding of Hsp70 and ARD1 after TNFα treatment (Supplementary Fig. 4B). We next constructed Flag-Hsp70 WT, K77R (deacetylation-mimic), and K77Q (acetylation-mimic) vectors and transiently transfected these plasmids into cells to study ARD1-mediated Hsp70 acetylation at K77 residues^24^. Flag-Hsp70 K77Q significantly decreased the phosphorylation level of S6K1 and the expression of p62 and partially restored LC3-II expression and the interaction between Vsp34 and Beclin-1 in ARD1-bearing cells after TNFα treatment (Fig. 4c). These results revealed that ARD-mediated Hsp70 acetylation inhibits mTOR activity and increases autophagy. Cell viability assays also revealed that the expression of Hsp70 WT and its mutants restrained the growth of ARD1-bearing cells after TNFα treatment (Supplementary Fig. 4C). Meanwhile, in contrast to Flag-Hsp70 WT and K77R, Flag-Hsp70 K77Q restricted cell growth in the presence of TNFα treatment (Supplementary Fig. 4D), suggesting that Hsp70-mediated autophagy is negatively related to cell survival in breast cancer.

### IKKβ promotes tumorigenesis by increasing mTOR activity and ARD1 degradation

**Fig. 5:**
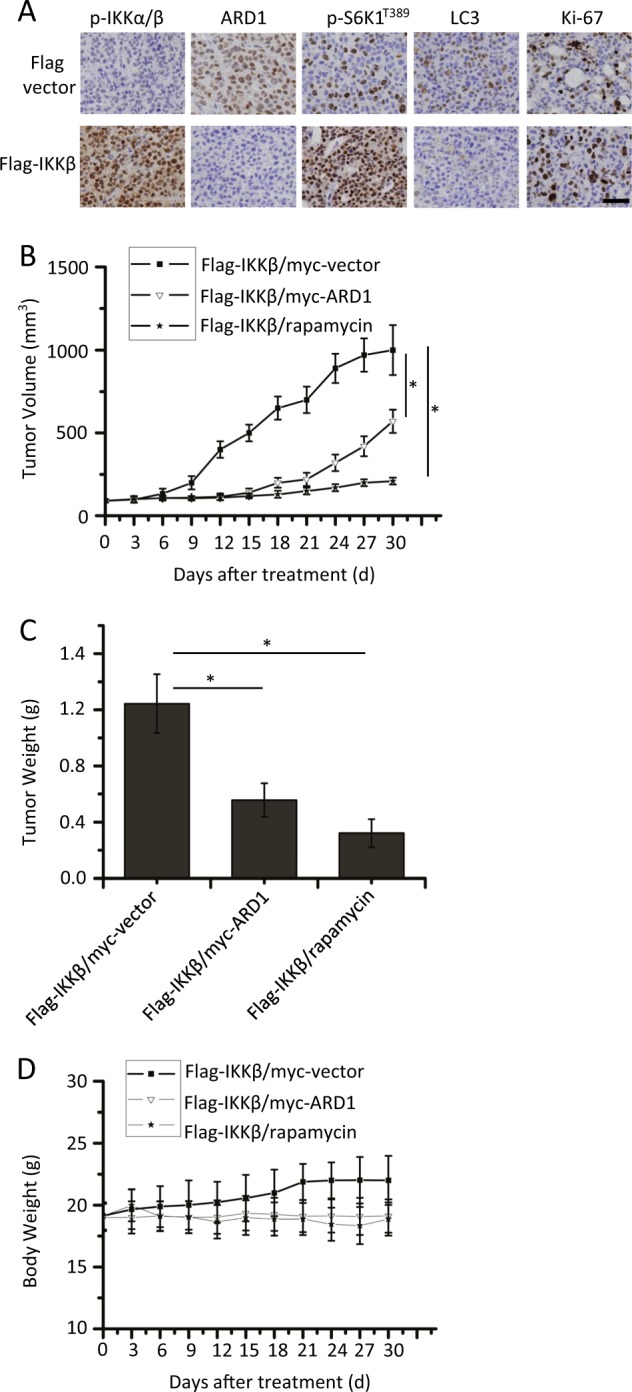
ARD1 decreased IKKβ mediated the growth of breast cancer in xenograft mice model. **a** Flag-IKKβ stably transfected MDA-MB-231 cells (1×10^6^) were injected into the mammary fat pads of BALB/c mice. At the end of experiments, tumor samples were examined for angiogenesis by Ki-67 immunostaining. p-IKKα/β, ARD1, LC3, or p-S6K1T^389^ expression was determined by immunohistochemical staining. Bars, 50 µM. **b** Flag-IKKβ/myc-vector or Flag-IKKβ/myc-ARD1 stably transfected MDA-MB-231 cells (1×10^6^) were injected into the mammary fat pads of BALB/c mice. Following tumor growth for 7 days, the tumor-bearing mice were randomly assigned into the following three groups (10 mice per treatment group): (a) Flag-IKKβ/myc vector, (b) Flag-IKKβ/myc-ARD1, and (c) Flag-IKKβ/ rapamycin group. For Flag-IKKβ/ rapamycin group, rapamycin (1.5 mg/kg) was injected into tumor-bearing BALB/c mice three times per week for 2 weeks and then tumor volumes were determined (*n* = 3, mean ± S.D. *P < 0.05). **c** Mice were treated as described in (**b**), and then the tumor weight of mice models was shown (*n* = 3, mean ± S.D. *P < 0.05). **d** Body weights of xenograft mice models were plotted at 3-day intervals. There were no significant differences in weights among the different groups (*P* > 0.05). Values were shown as mean ± S.D.

We next determined whether IKKβ-mediated ARD1 processing contributes to breast cancer tumorigenesis. We stably transfected Flag-IKKβ plasmids into MDA-MB-231 cells and injected these cells into nude mice to establish xenograft tumors. IKKβ overexpression increased the phosphorylation levels of IKKα/β and S6K1. Meanwhile, ARD1 and LC3 expression in the tumors was decreased with overexpression of IKKβ (Fig. 5a).

We also constructed cells with stable Flag-IKKβ/myc-vector and Flag-IKKβ/myc-ARD1 expression. These cells were injected into mice to form the corresponding tumors. Tumors expressing IKKβ and ARD1 grew more slowly than those with only IKKβ. Meanwhile, rapamycin inhibited IKKβ-mediated tumor progression (Fig. 5b) as described before^5^. Similar results were also found for tumor weight (Fig. 5c). To evaluate the possible adverse effects of plasmid transfection or rapamycin injection, the weight of the mice was monitored every 3 days throughout the whole experiment. The body weights of the different groups were similar (Fig. 5d) and no ruffled fur or toxic death was observed in the different groups.

## Discussion

The role of IKKβ in cancer procession is complex. IKKβ can both promote and prevent carcinogenesis depending on the cell type or molecular context^25–28^. Even in intestinal tumorigenesis, IKKβ was reported to play an opposite role^27,28^. However, in the development of breast cancer, we and other research groups have shown that IKKβ plays an important role in promoting the occurrence of cancer^5,29,30^. The same scenario also happened with ARD1, as the role of ARD1 in tumor development is also controversial. But such as IKKβ, the role of ARD1 in breast cancer is clear. ARD1 was reported to inhibit mTOR activity to limit breast cancer cell growth^19^. Meanwhile, in a xenograft mouse model, ARD1 overexpression decreased xenograft breast cancer growth^18^, indicating that ARD1 is a suppressor of breast tumorigenesis. Meanwhile, ARD1 was reported to be phosphorylated and degraded by IKKβ, thereby relieving the inhibition of cell growth^6^. These studies prompted us to think about whether there is an interaction between IKKβ and the ARD1 pathway. Fortunately, we found that IKKβ mediates ARD1 degradation during the process of TNFα-induced cell growth of breast cancer. In this way, IKKβ regulates cell growth through two pathways. On one hand, IKKβ directly acts on TSC1^5^, thereby relieving the inhibition of mTOR activity; on the other hand, IKKβ indirectly acts on TSC2 and promotes mTOR activity via degradation of ARD1^19^. This increased mTOR activity promotes cell growth.

In this study, we first demonstrated that IKKβ inhibits an ARD1-mediated autophagy pathway. Moreover, we have showed that acetylation of Hsp70 is also involved in ARD1-mediated autophagy. Hsp70, an important chaperone protein in protein folding and assembly, is highly expressed in tumor cells where it promotes the growth of tumor cells^24^. Hsp70 acetylation at K77 mediates the balance of protein refolding and degradation, to promote cell survival and limit cell death stress^24^. Meanwhile, Hsp70 acetylation at K77 inhibits autophagic cell death in cancer cells;^31^ however, these research results are somewhat different from ours.

In our study, Hsp70 acetylation-mediated autophagy decreased cancer cell growth. Moreover, the reduction of Hsp70 acetylation was induced by a cell survival factor in our experiments. Our research revealed an inhibitory role of Hsp70 acetylation-mediated autophagy during breast cancer cell growth after TNFα treatment, whereas recent studies have demonstrated that autophagy mediated by Hsp70 acetylation maintains cell growth after the treatment with a cell death factor^24,31^. These results indicate that Hsp70 and its mediated autophagy play different functions under different conditions and in different tumor cells. After all, Hsp70 can combine with Hsp90, Hop, CHIP, and other proteins to form complexes and can play different roles at different times^24^. The effect of this complex-mediated autophagy in tumorigenesis is unclear^32^. Moreover, Hsp70 acetylation in breast cancer cells was indeed reported to inhibit cell growth^33,34^.

Our work revealed that ARD1 acetylated Hsp70 to inhibit breast cancer cell growth. Meanwhile, ARD1 also acetylated and stabilized TSC2 to decrease breast cancer cell growth^19^. Previous work demonstrated that ARD1 acetylated β-catenin to promote lung cancer cell proliferation^9^. These results might suggest that ARD1 may play different functions in different cell types by associating with and acetylating various substrates. Moreover, analyzation of the amino acid sequence of ARD1 shows that it contains several potential phosphorylation sites. In addition to the Ser209 locus, it will be of great interest to determine whether other phosphorylation/dephosphorylation events are involved in ARD1 regulation, thereby mediating ARD1’s functions in different cancer development. Of course, further investigation is needed to clarify whether ARD1 can act as a tumor suppressor or an oncoprotein or can have a role in both capacities in different cancer types or under different conditions.

In summary, we elaborated on the detailed mechanisms of IKKβ-mediated breast cancer cell growth and confirmed that ARD1-induced autophagy contributes to IKKβ-mediated breast cancer tumorigenesis. These findings have important implications for the clinical treatment of breast cancer.

## Materials and methods

### Materials

Flag (F7425), actin (clone AC-74, A5316) antibodies, and 3-MA (M9281) were supplied by Sigma (St. Louis, MO, USA). HA (11666606001) and myc (11667203001) antibodies were obtained from Roche ((Roche Applied Science, Laval, PQ, Canada). TSC2 (SC-893), Beclin-1 (E-8) (sc-48341), S6K1 (SC-230) antibodies, and TNFα (SC-8301) were purchased from Santa Cruz Biotechnology (Santa Cruz, CA, USA). p-S6K1^T389^ (9206 and 9234), p-4EBP1^S65^ (9451), p53 (clone 7F5, 2527), p62 (88588), IKKα (2682), IKKβ (2684), p-IKKα/β (2697), ARD1 (13357), LC3 (3868), Hsp70 (4872), acetylated lysine (acetyl-lys, 9441) antibodies, and MG132 (2194) were purchased from Cell Signaling Technology (Beverly, MA, USA). Vps34 (AP8014a) antibody was purchased from Abgent (San Diego, CA, USA). ARD1 antibody for immunohistochemical staining was a gift from Chengchao Shou (Peking University, Beijing, China)^18^.

### Plasmid construction and RNAi

Full-length human IKKα, IKKβ, and Hsp70 cDNAs were cloned with N-terminal FLAG epitope into pcDNA3. Full-length IKKα and IKKβ are the gifts from Claus Scheidereit (Max Delbru¨ck Center for Molecular Medicine, Berlin, Germany). Meanwhile, kinase-dead mutants of IKKα and IKKβ carry a K44A mutation (nIKKα and nIKKβ) vectors are also from Claus Scheidereit. We constructed Myc-ARD1- and HA-ARD1-expressing plasmids by inserting hARD1 complementary DNA (cDNA) into pcDNA6-myc and pCMV5-HA vectors, respectively. The pCDNA6-Myc-Rheb and pCMV5-HA-TSC2 constructs were the kind gifts from Kun-Liang Guan (University of Michigan Medical School, MI, USA). The pRK7-HA-S6K1 plasmid was from J. Blenis (Harvard Medical School, Boston, MA, USA) and pACTAG2-3HA-4EBP1 was from N. Sonenberg (McGill University, Montreal, Canada). GFP-LC3 construct was a gift from Quan Chen (Chinese Academy of Sciences, Beijing, China). The ARD1 or Hsp70 mutant was generated by site-directed mutagenesis using Pfu-ultra poly-merase (Stratagene, La Jolla, CA, USA) followed by *Dpn*I digestion (Fermentas Inc., Glen Burnie, MD, USA) according to the manufacturer’s instructions. The following small interfering RNAs (siRNA; siGenome SMARTpool) were obtained from Dharmacon (Lafayette, CO, USA) as a pool of four annealed double-stranded RNA oligonucleotides: IKKα (M-003473-02), IKKβ (M-040630-00), ARD1 (J-049547), or sicontrol not targeting pool siRNAs (D-001810-10-20).

### Cell culture and transfection

MCF-7, MDA-MB-231, and HEK293T cell lines were from the American Type Culture Collection (ATCC). MDA-MB-231, HEK293T, and MEFs were cultured in DMEM with 10% fetal bovine serum (FBS). TSC2^+/+^/p53^−/−^ and TSC2^−/−^/p53^−/−^ MEFs from David J. Kwiatkowski (Brigham and Women’s Hospital, Boston, USA). MCF-7 cells were cultured in IMEM, 10% FBS, and 10 μg/ml insulin. Plasmids were transfected into cells using Amaxa nucleofector kits (Lonza Inc., Allendale, NJ, USA), and then cells were treated. Treated cells were subjected to western blot analysis. For stable transfection, cells were selected by G418 (pcDNA3-Flag) or blasticidin S (pcDNA6-myc), respectively.

### Assay of cell viability and proliferation and GFP- LC3 assay

For assay of cell viability and proliferation, treated cell viability was detected by CellTiter-Glo Luminescent Cell Viability Assay from Promega as described before^35,36^. GFP- LC3 assay was performed in MCF-7 cells transiently transfected with GFP-LC3 constructs, and then the cells were cultured on coverslips and then treated for the indicated times. Fluorescence was immediately observed using an Olympus DP72 microscope (Olympus Corporation, Tokyo, Japan).

### Immunoprecipitation and western blot analysis

For immunoprecipitation, all cells were harvested by resuspension in CHAPS cell extract buffer (Cell Signaling) and sonicated on ice. Lysates were centrifuged at 14,000×*g* at 4 °C for 15 min. Cell extracts were precleared and incubated with antibodies against ARD1, IKKα, Beclin-1, HA, myc, or Hsp70 with protein A-Sepharose (Invitrogen) to pull down immune complexes. The Sepharose was washed three times with lysis buffer and two times with PBS. Total lysates and immunoprecipitates were analyzed by western blot^37,38^.

### In vivo tumor experiments

Animal studies were approved by the Institutional Animal Care and Treatment Committee of Sichuan University (Chengdu, China) and performed along established institutional animal welfare guidelines concordant with the United States guidelines (NIH Publication #85-23, revised in 1985). We performed tumorigenesis assays in an orthotopic breast cancer mouse model^39^. In brief, 1 × 10^6^ different MDA-MB-231 cell lines were subcutaneously injected into the mammary fat pad of 6- to 8-week-old female athymic nude BALB/c mice (Vital-River Laboratories, Beijing, China; ten mice per group). Tumor volumes were evaluated according to the following formula: tumor volume (mm^3^) = 0.52 × length × width^2^. The weight of the mice was measured at 3-day intervals. At the end of the experiment, the mice were killed. Tumor net weight of each mouse was measured. Angiogenesis was examined by Ki-67 immunostaining. Protein expression was measured by immunohistochemical staining as described before^18,35^.

### Statistical analysis

The statistical analysis was performed with SPSS software (version 17.0 for Windows). Results are presented as mean ± S.D. Analysis of variance and the Tukey–Kramer multiple-comparison test were used in comparisons. *P* < 0.05 was considered statistically significant.

## Electronic supplementary material

Supplementary Figure legends

Supplementary Figure 1

Supplementary Figure 2

Supplementary Figure 3

Supplementary Figure 4-1

Supplementary Figure 4-2
