# Supplementary material for: ARD1 contributes to IKKβ-mediated breast cancer tumorigenesis
Source: Cell Death Dis. 2018 Aug 28;9(9):860. doi: 10.1038/s41419-018-0921-2 (PMC6113314; doi:10.1038/s41419-018-0921-2)
Supplement: Supplementary file 1 — Supplementary Figure legends [file 41419_2018_921_MOESM1_ESM.docx]

**Supplementary Figure legends**

**Supplementary Figure 1. A.** MCF-7 and MDA-MB-231 cells were serum-starved overnight and treated with TNFα (10 ng/ml) and/or MG132 (10 μM) for 1 h. Treated cells were collected for western blot analysis of the expression of protein shown in the figure. β-Actin was used as a protein loading control. **B.** Exogenous interaction of ARD1 and IKKβ. HEK293T cells were cotransfected with HA-ARD1, FLAG-IKKβ, FLAG-nIKKβ or FLAG-IKKα, FLAG-nIKKα, and then cell lysates were immunoprecipitated with anti-FLAG or anti-HA antibodies. The association between HA-ARD1 and FLAG-IKKβ or FLAG-nIKKβ was analyzed by coimmunoprecipitation and immunoblotting assays. **C.** Cells were transfected with Ctrl siRNA, IKKβ or IKKα for 48 h, and then cells were collected for western blot analysis of the expression of protein shown in the figure. **D.** Cells were cotransfected with FLAG-IKKβ, HA-ARD1 WT or HA-ARD1 mutant for 48 h and cells were collected for detection of cell viability and proliferation used by CellTiter-Glo Luminescent Cell Viability Assay. Graphs showing results of quantitative analyses (*n*=3, mean ± S.D. *, *P*<0.05).

**Supplementary Figure 2. A.** Indicated cells were transfected with Ctrl siRNA, ARD1 siRNA for 48 h, serum-starved overnight, and then treated with TNFα for 1 h. Treated cells were collected for western blot analysis of the expression of protein shown in the figure. β-Actin was used as a protein loading control. **B.** HEK293T cells were cotransfected with FLAG-IKKβ, Myc-ARD1 and HA-TSC2. Meanwhile, transfected cells were treated by MG132 (10 μM). Treated cells were lysed for coimmunoprecipitation assay as described**. C.** MCF-7 cells were serum-starved overnight and treated with TNFα and MG132 (10 μM) for 1 h. Cell lysates were immunoprecipitated with specific antibodies to ARD1 and TSC2 to identify the association of endogenous proteins. **D.** Lysates of indicated cells were collected with western blot analysis for detection of TSC2 and p53 expression in cells. β-Actin was used as a protein loading control.

**Supplementary Figure 3. A.** Cells were transfected with Ctrl siRNA or IKKβ siRNA for 48 h, serum-starved overnight, and then treated with TNFα for 1 h. Cell lysates were collected for detection of LC3-I and LC3-II expression. β-Actin was used as a protein loading control. **B.** MCF-7 cells were cotransfected with Ctrl siRNA or ARD1 siRNA and myc-Rheb. Cell lysated were collected for LC3-I and LC3-II expression detection. **C.** Cells were transfected with Ctrl siRNA or ARD1 siRNA and then treated with the inhibitor of class III PI3 kinases 3-MA (5 mM) for 4 h, lysed and subjected to western blotting with anti-LC3 antibodies to monitor autophagy. **D** and **E.** Cells were treated as described in **B** and **C**, respectively. Cell viability and proliferation were determined. Graphs showing results of quantitative analyses (*n*=3, mean ± S.D. *, *P*<0.05).

**Supplementary Figure 4. A.** Cells were transfected with Ctrl siRNA or ARD1 siRNA and then the serum-starved overnight. Cells were treated with TNFα for 30 mins. Cell lysates were detected by immunoprecipitation as described in the figure. **B.** Cells were serum-starved overnight. Cells were treated with TNFα and MG132 (10 μM) for 30 mins. Cell lysates were detected as described in **A**. **C.** Cells were cotransfected with HA-ARD1 or Flag Ctrl, Flag vector, Flag-Hsp70 WT, Flag-Hsp70 mutants, serum-starved overnight, and then treated with TNFα for 1 h. Cell viability and proliferation were determined. Graphs showing results of quantitative analyses (*n*=3, mean ± S.D. *, *P*<0.05). **D.** Cells were cotransfected with Flag Ctrl, Flag vector, Flag-Hsp70 WT or Flag-Hsp70 mutants, and then treated as **C.** Graphs showing results of quantitative analyses (*n*=3, mean ± S.D. *, *P*<0.05).
