# Supplementary figures and images for: ARD1 contributes to IKKβ-mediated breast cancer tumorigenesis
Source: Cell Death Dis. 2018 Aug 28;9(9):860. doi: 10.1038/s41419-018-0921-2 (PMC6113314; doi:10.1038/s41419-018-0921-2)

Supplementary Fig.1

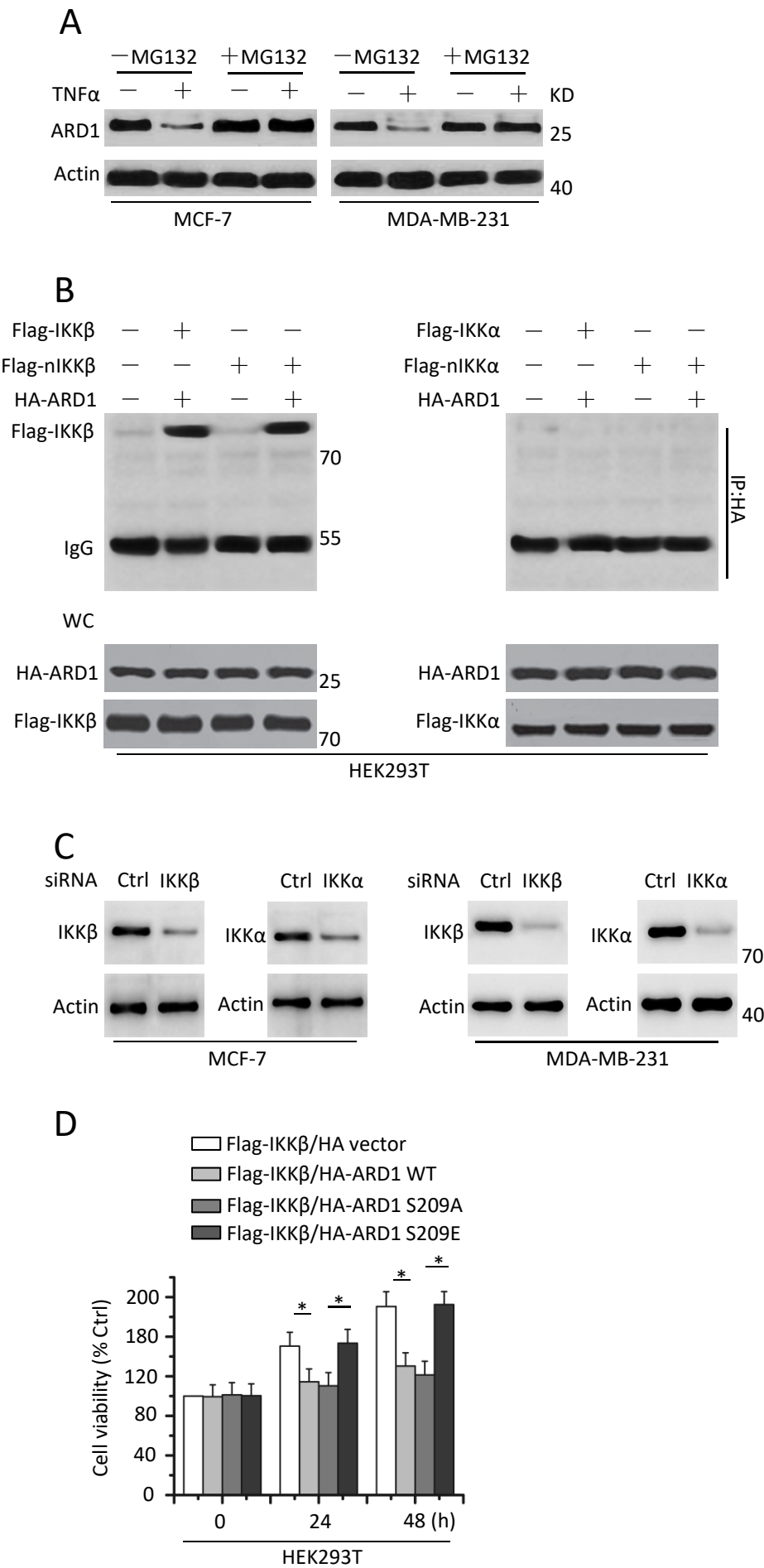

Supplement: Supplementary file 2 — Supplementary Figure 1 [file 41419_2018_921_MOESM2_ESM.pdf]

Supplementary Fig.2

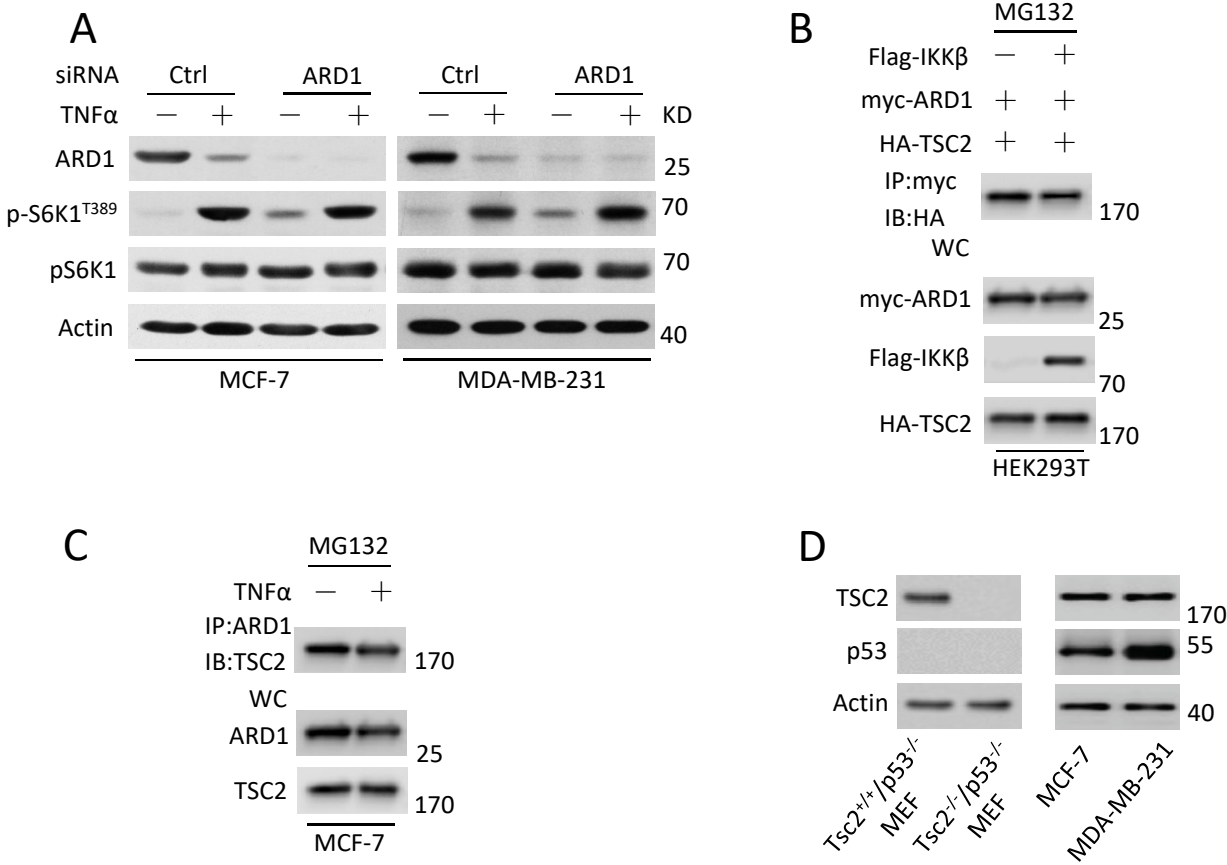

Supplement: Supplementary file 3 — Supplementary Figure 2 [file 41419_2018_921_MOESM3_ESM.pdf]

Supplementary Fig.3

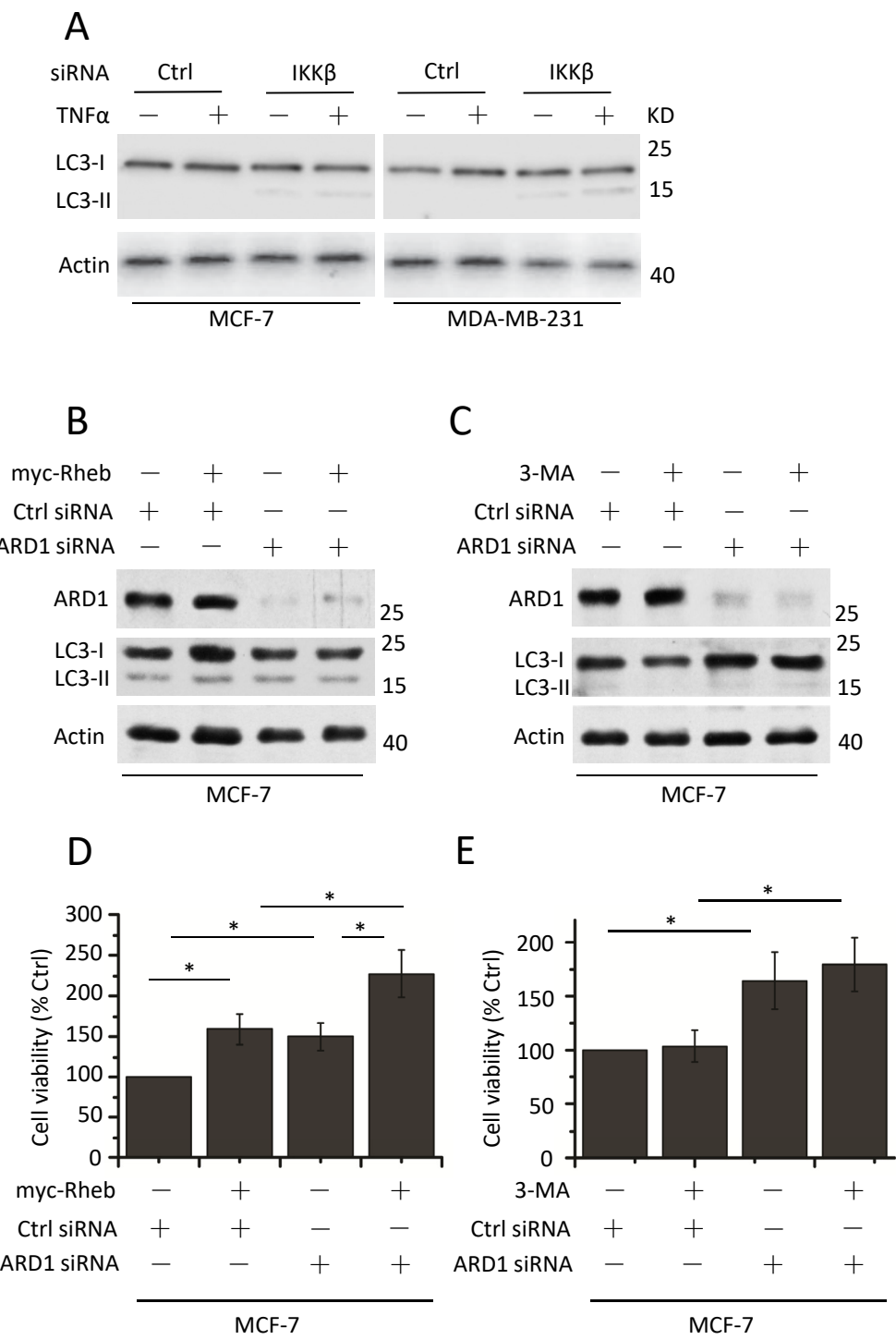

Supplement: Supplementary file 4 — Supplementary Figure 3 [file 41419_2018_921_MOESM4_ESM.pdf]

Supplementary Fig.4

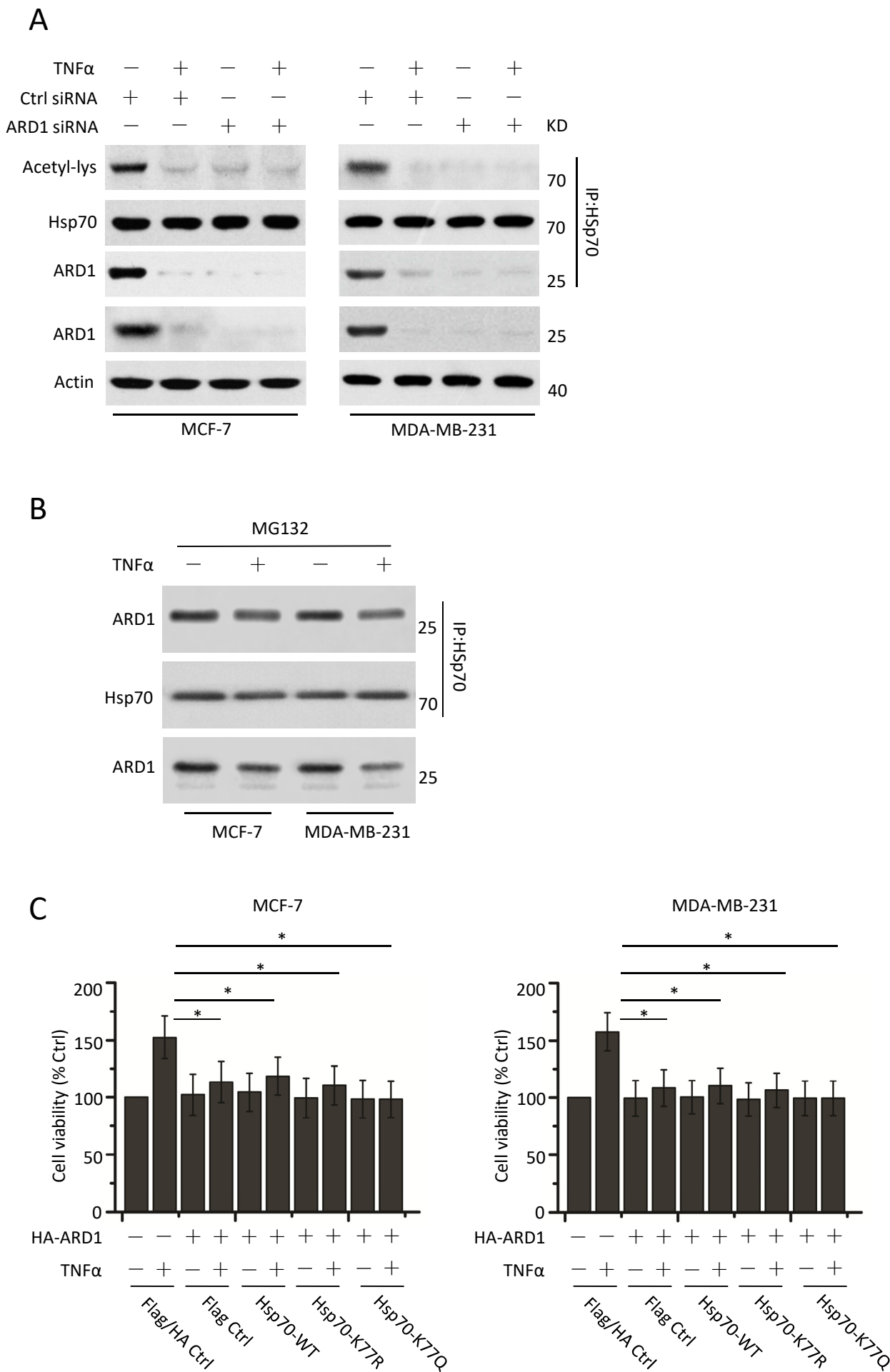

Supplement: Supplementary file 5 — Supplementary Figure 4-1 [file 41419_2018_921_MOESM5_ESM.pdf]

Supplementary Fig.4

D

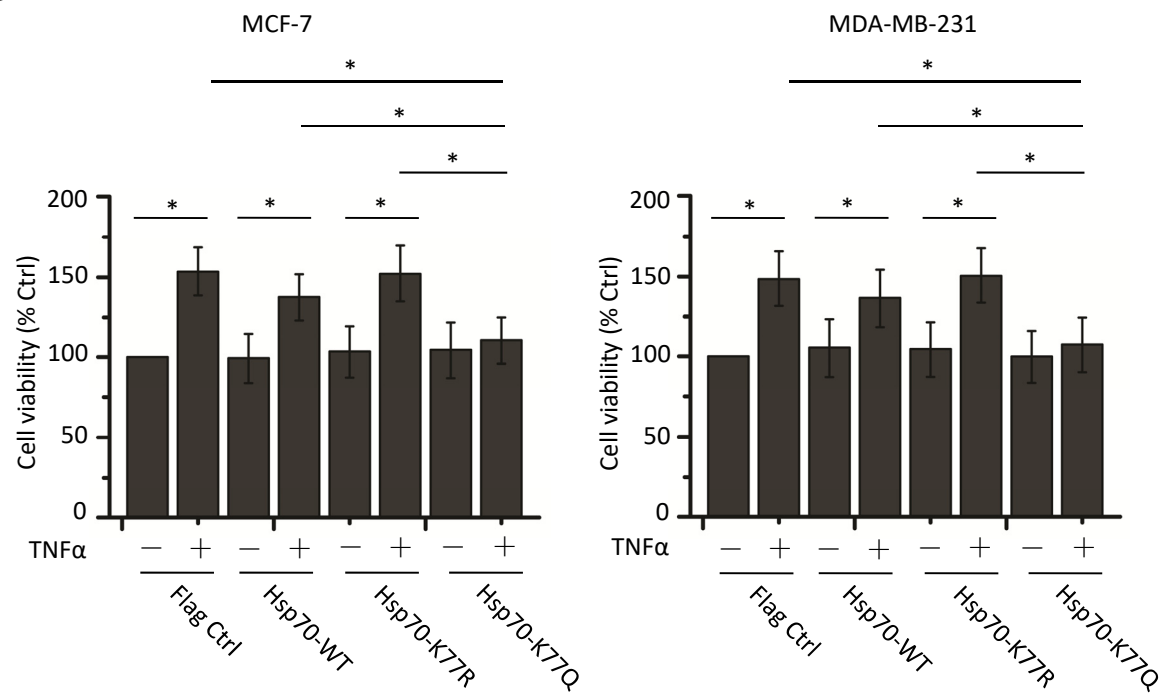

Supplement: Supplementary file 6 — Supplementary Figure 4-2 [file 41419_2018_921_MOESM6_ESM.pdf]
